# Supplementary material for: Initiator-Directed Transcription: Fission Yeast Nmtl Initiator Directs Preinitiation Complex Formation and Transcriptional Initiation
Source: Genes (Basel). 2022 Jan 28;13(2):256. doi: 10.3390/genes13020256 (PMC8871863; doi:10.3390/genes13020256)
Supplement: Supplementary file 1 [file genes-13-00256-s001.zip › Figure S1.pdf]

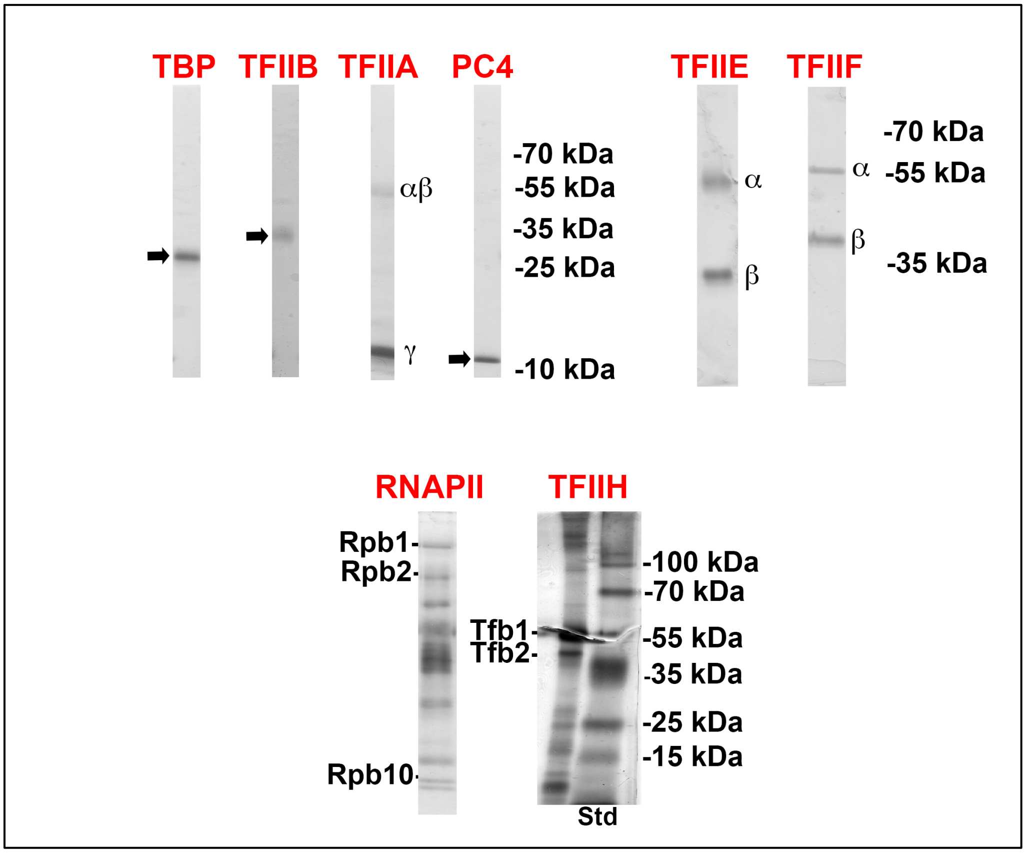

<u>Figure S1:</u> Silver stained SDS-PAGE of purified GTFs and PC4 used in the work. TBP, TFIIB, TFIIA, PC4, RNAPII and TFIIH were analyzed in a gradient gel (4 – 14% polyacrylamide). TFIIE and TFIIF were analyzed in a 10% SDS-PAGE. Arrow indicates the position of the protein band associated to each TBP, TFIIB or PC4. α, β and γ indicates the subunits of TFIIA, TFIIE and TFIIF factors.
